# Human Metapneumovirus Detection in Patients with Severe Acute Respiratory Syndrome

**DOI:** 10.3201/eid0909.030304

**Published:** 2003-09

**Authors:** Paul K.S. Chan, John S. Tam, Ching-Wan Lam, Edward Chan, Alan Wu, Chi-Kong Li, Thomas A. Buckley, King-Cheung Ng, Gavin M. Joynt, Frankie W.T. Cheng, Ka-Fai To, Nelson Lee, David S.C. Hui, Jo L.K. Cheung, Ida Chu, Esther Liu, Sydney S.C. Chung, Joseph J.Y. Sung

**Affiliations:** * Faculty of Medicine of the Chinese University of Hong Kong, Prince of Wales Hospital, Shatin, New Territories, Hong Kong SAR, China

**Keywords:** Metapneumovirus, coronavirus, severe acute respiratory syndrome, SARS, virus isolation, Hong Kong, China

## Abstract

We used a combination approach of conventional virus isolation and molecular techniques to detect human metapneumovirus (HMPV) in patients with severe acute respiratory syndrome (SARS). Of the 48 study patients, 25 (52.1%) were infected with HMPV; 6 of these 25 patients were also infected with coronavirus, and another 5 patients (10.4%) were infected with coronavirus alone. Using this combination approach, we found that human laryngeal carcinoma (HEp-2) cells were superior to rhesus monkey kidney (LLC-MK2) cells commonly used in previous studies for isolation of HMPV. These widely available HEp-2 cells should be included in conjunction with a molecular method for cell culture followup to detect HMPV, particularly in patients with SARS.

Human metapneumovirus (HMPV) was first identified in 2001 in samples from children with respiratory tract diseases (*1*). Subsequent studies showed that the virus is responsible worldwide for a proportion of community-acquired acute respiratory tract infections in children (*2*–*4*), as well as other age groups (*5*–*9*). Co-infection of HMPV with respiratory syncytial virus (RSV) in infants has been suggested to be a factor that influences the severity of bronchiolitis (*10*).

HMPV is a new member of the family *Paramyxoviridae*, subfamily *Pneumovirus*. The overall percentage of amino acid sequence homology between HMPV and avian metapneumovirus (APV) ranges from 56% to 88% for open reading frames N, P, M, F, M2-1, M2-2, and L (*11*). Phylogenetically, RSV is the closest human virus related to HMPV, and the clinical symptoms of HMPV may share an overlapping spectrum with RSV (*2*,*4*,*7*,*9*,*10*). The epidemiology and symptoms of HMPV infection have not been fully elucidated; one obstacle in establishing these data is the difficulty in establishing a laboratory diagnosis of the infection. We describe our experience of detecting HMPV during an outbreak of severe acute respiratory syndrome (SARS).

## Methods

### Study Population

In early March 2003, an outbreak of SARS occurred in the Prince of Wales Hospital (the teaching hospital of The Chinese University of Hong Kong). Our study participants were patients admitted to our hospital for suspected SARS during the first week of the outbreak (*12*). These patients fulfilled the World Health Organization definition for probable SARS cases (*13*). Briefly, patients had an acute onset of fever (>38°C, most with chills or rigor), dyspnea, myalgia, headache, and hypoxemia. Peripheral air-space consolidation subsequently developed in all study patients as observed on chest radiographs or thoracic computed tomographic scan; patients showed no response to antimicrobial drugs prescribed for typical and atypical pneumonia (β-lactams, macrolides, and fluoroquinolones).

During our study, we examined 48 patients who comprised our first group of SARS patients and had a clear history of exposure. Forty-five participants were adults (26 men, 19 women) 21–69 years of age (mean 35.4 years of age; standard deviation 11.5 years). The group included 26 healthcare workers and 7 medical students who worked in a ward (index ward) in the hospital where a few patients with SARS had stayed. The remaining 12 patients had been hospitalized or were visitors to the same ward. Three study participants were children (two boys, one girl) 2–7 years of age. All these children were living with persons who had been hospitalized or were visitors to the index ward and who had contracted SARS.

### Virus Isolation

Nasopharyngeal aspirate (NPA) samples were taken from all patients by inserting a suction catheter into the nasopharyngeal area via the nostril. A low suction force was applied to collect approximately 0.5 mL fluid, which was then transferred into 2 mL of viral transport medium. All NPAs were added onto rhesus monkey kidney (LLC-MK2), human laryngeal carcinoma (HEp-2), Mardin Darby Canine Kidney (MDCK), human embryonic lung fibroblast, Buffalo green monkey kidney (BGM), and African green monkey kidney (Vero) monolayers. All cell cultures were incubated at 37°C, except for MDCK, which was incubated at 33°C. All NPAs were added to an additional LLC-MK2 cell culture tube and incubated at 33°C. Cell monolayers were examined daily for cytopathic effect. After 14 days of incubation, a hemadsorption test for LLC-MK2 and MDCK monolayers was performed. All cell cultures materials were kept frozen for subsequent analyses.

### Human Metapneumovirus Reverse Transcription-Polymerase Chain Reaction (RT-PCR)

To detect HMPV, we used a nested RT-PCR focused on the F-gene. This RT-PCR was applied on all cell cultures, regardless of cytopathic effect. After one cycle of freeze-and-thaw, RNA was extracted from cell cultures by using the QIAamp Viral RNA Mini Kit (QIAGEN GmbH, Hilden, Germany), according to the manufacturer’s protocol. The outer primers were 5′-AGC TGT TCC ATT GGC AGC A-3′ for RT and amplification and 5′-ATG CTG TTC RCC YTC AAC TTT-3′ (R = A or G, Y = C or T) for amplification. These primers were designed on the basis of HMPV sequences available from GenBank. The reaction was carried out in a single-tube (Superscript One-Step RT-PCR and Platinum Taq; Invitrogen Corp., Carlsbad, CA) by using 0.2 μM of each primer and thermal cycling conditions of 50°C for 30 min and 94°C for 3 min; followed by 40 cycles of 94°C for 30 s, 52°C for 30 s, 72°C for 45 s, and a final extension at 72°C for 7 min. For the second round of amplification, we used 0.2 μM of inner primers 5′-GAG TAG GGA TCA TCA AGC A-3′ and 5′-GCT TAG CTG RTA TAC AGT GTT-3′. The PCR was conducted at 95°C for 15 min for denaturation of DNA templates and activation of the hot-start DNA polymerase (HotStarTaq, QIAGEN GmbH), followed by 40 cycles at 94°C for 30 s, 54°C for 30 s, and 72°C for 45 s, and a final extension at 72°C for 7 min. PCR products detected by agarose gel electrophoresis were analyzed for sequence homology with known HMPV strains. In addition to virus isolation, RNA was extracted directly from NPAs for HMPV RT-PCR by using the same protocol as for cell cultures.

### Coronavirus RT-PCR

RNA was extracted from the supernatant of Vero cell cultures showing cytopathic effect by using the same method as for HMPV. Coronavirus was detected by RT-PCR with primers COR-1 (sense) 5′ CAC CGT TTC TAC AGG TTA GCT AAC GA 3′ and COR-2 (antisense) 5′ AAA TGT TTA CGC AGG TAA GCG TAA AA 3′, which had been shown to be specific for the novel coronavirus detected from patients with SARS (*14*). The RT-PCR for coronavirus was conducted similarly to HMPV (by using 0.6 μM of each primer and thermal cycling conditions of 54°C for 30 min, 94°C for 3 min; 45 cycles of 94°C for 45 s, 60°C for 45 s, 72°C for 45 s; and 72°C for 7 min).

### Sequence Analysis

The nucleotide sequence of purified PCR products was determined by PCR-based cycle sequencing performed with the inner primers. Sequencing reactions were performed according to the manufacturer’s protocol (BigDye Terminator Cycle Sequencing Kit version 3.1, Applied Biosystems, Foster City, CA) and run on the ABI Prism 3100 Genetic Analyzer. All sequences were confirmed by repeated PCRs and sequencing from both directions.

### Electron Microscopy

Selected cell cultures that showed cytopathic effect were examined by electron microscopy. Cell culture supernatants were coated on formvar-carbon grids and stained with 2% phosphotungstic acid.

### Antibody Detection

To ascertain the HMPV culture results, we obtained paired serum samples (first sample collected within 5 days and second sample collected >14 days after onset of illness) and tested for HMPV antibody. HMPV–infected LLC-MK2 cells were coated on 12-well glass slide and fixed in acetone. The presence of antibody in serum samples was tested for by using the direct immunofluorescence technique.

### Exclusion of Cross-Contamination and Test for Reproducibility

Specimen processing, viral culture inoculation, RNA extraction, RT-PCR amplification, and PCR product analyses were conducted in different rooms. Special care was taken to avoid contamination with RNase, and to avoid cross-contamination between reactions. During the inoculation of cell monolayers, we placed a negative control using the same cell line injected with maintenance medium after every fifth cell culture tube. These negative control cell culture tubes were also incubated, examined for cytopathic effect, and processed for RT-PCR as for cell culture tubes injected with specimens. For RNA extraction and RT-PCR procedures, we placed negative controls using cell culture medium to replace cell supernatant injected with NPAs or double distilled water to replace NPA sample after every fifth reaction. These negative controls did not show positive results, which indicated the absence of cross-contamination. To test the reproducibility of RT-PCR results, we repeated the testing of all positive samples and 30 randomly selected negative samples; all results were reproducible. We also spiked negative NPA samples with HMPV RNA and repeated the extraction and RT-PCR procedures. The results showed no inhibitors were present in the extracted RNA preparations.

## Results

Of the 48 NPAs studied, we observed no cytopathic effect on HEp-2, MDCK, human embryonic lung fibroblast, and BGM monolayers. Eleven (22.9%) specimens showed cytopathic effect of diffuse refractile rounding of cells on Vero cell monolayers 2–4 days after incubation, progressed rapidly, and involved the whole monolayer within 12–24 hours. The same cytopathic effect was reproducible on passage to Vero cells, and appeared 1 to 2 days after incubation. These Vero cell cultures were all positive by the coronavirus RT-PCR. The Vero cell culture supernatants showing cytopathic effect were randomly selected for electron microscopy examination, and coronavirus particles were seen.

Five specimens showed cytopathic effect of focal refractile rounding of cells in LLC-MK2 monolayers. All these LLC-MK2 cell cultures had been incubated at 37°C. Unlike the cytopathic effect observed in Vero cells attributable to coronavirus, this cytopathic effect developed after 10 to 12 days of incubation and progressed slowly to detachment from the cell monolayer.

The HMPV RT-PCR examination of cell cultures was negative for human embryonic lung fibroblast, BGM cells, and Vero cells (including those positive for coronavirus). In contrast, HMPV RT-PCR showed a PCR product of the expected size (89 bp) from 25 (52.1%) isolation materials injected with specimens. The nucleotide sequences of the PCR products were identical to the F-gene fragment of HMPV (GenBank accession no. NC 004148) (*1*). We retrospectively examined the first round of PCR products of all positive samples. Those positive samples derived from direct NPAs did not show positive band, indicating a nested RT-PCR was necessary. However, most (27 [90%] of 30) of those derived from cell cultures showed a positive band of the expected size from the first round of PCR. The distribution of HMPV RT-PCR results on direct detection of NPAs and from different cell culture types is shown in the Table. Overall, the sensitivity of direct detection of NPAs using HMPV RT-PCR was 2 (8.0%) of 25 samples. In one of these two samples, we isolated the virus from three cell lines. In the other sample, we isolated virus from HEp-2 and LLC-MK2. Overall, HEp-2 was the most sensitive cell lines (22 [88.0%] of 25 HMPV positive samples); LLC-MK2 cells detected 6 (24.0%) of 25 samples, and MDCK cells detected 2 (8.0%) of 25 samples. Most (with the exception of three LLC-MK2–positive samples) showed positive results in HEp-2 cells. All six LLC-MK2 cell cultures positive for HMPV were incubated at 37°C; three of these positive cultures that had had the corresponding LLC-MK2 cell cultures incubated at 33°C showed positive results.

**Table:** Distribution of human metapneumovirus reverse transcription–polymerase chain reaction results among 25 positive nasopharyngeal aspirates^a,b^

No. of patients (%)	Human metapneumovirus F-gene sequence detected by RT-PCR			
	Nasopharyngeal aspirate	HEp-2 cells	LLC-MK2 cells	MDCK cells
1 (4.0)	Positive	Positive	Positive	Positive
1 (4.0)	Positive	Positive	Positive	Negative
1 (4.0)	Negative	Positive	Positive	Negative
1 (4.0)	Negative	Positive	Negative	Positive
18 (72.0)	Negative	Positive	Negative	Negative
3 (12.0)	Negative	Negative	Positive	Negative

^a^RT-PCR, reverse transcription-polymerase chain reaction; Hep-2, human laryngeal carcinoma monolayer; LLC-MK2, rhesus monkey kidney monolayer; MDCK, Mardin Darby Canine Kidney monolayer; BGM, Buffalo green monkey kidney monolayer. ^b^The human metapneumovirus RT-PCR results for all human embryonic lung fibroblast, BGM and Vero cell cultures were negative.

**Figure 1:**
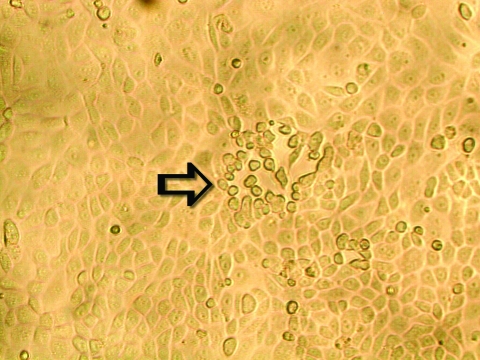
Early cytopathic effect of human metapneumovirus in rhesus monkey kidney (LLC-MK2) cell monolayers. A focus of infected cells that exhibit refractile rounding is indicated by an arrow (100X).

**Figure 2:**
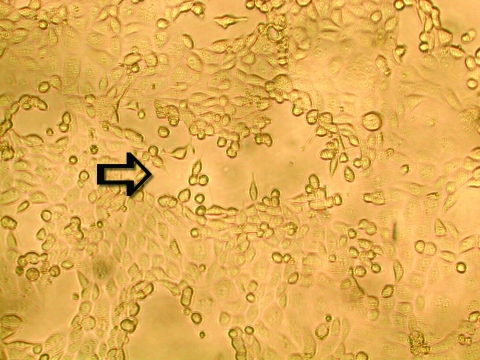
Late cytopathic effect of human metapneumovirus in rhesus monkey kidney (LLC-MK2) cell monolayers. Infected cells progressed slowly from focal rounding to detachment from cell monolayer is indicated by an arrow (100X).

**Figure 3:**
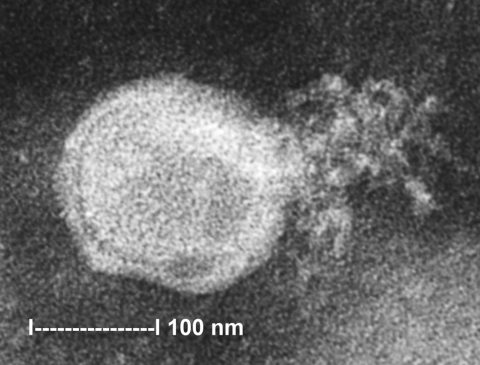
Electron micrograph of human metapneumovirus collected from the supernatant of rhesus monkey kidney (LLC-MK2) cell culture. A virion releasing nucleocapsid is shown.

To ascertain that cell cultures with HMPV RT-PCR–positive results represented the isolation of HMPV, all LLC-MK2 (incubated at 37°C), HEp-2, and MDCK cell cultures, regardless of the HMPV RT-PCR findings, were passaged to LLC-MK2 cells for a prolonged incubation of 28 days. HEp-2 cells were not used for this purpose because HEp-2 cell monolayers are often difficult to maintain for >2 weeks. The results showed that all passages from HMPV RT-PCR–positive cell cultures showed cytopathic effect of focal refractile rounding of cells that occurred after 10 to 22 days of incubation (Figure 1). The cytopathic effect progressed slowly to detachment from the cell monolayer (Figure 2). The supernatants of these passages were also positive by the HMPV RT-PCR and had visible HMPV viral particles on electron microscopy examination (Figure 3). The passages from HMPV RT-PCR–negative supernatants did not show positive results by the above tests. We also passaged five Vero cell cultures that were positive for coronavirus to LLC-MK2 cells in a similar way. All of these passages did not show cytopathic effect and were negative by the HMPV RT-PCR.

To reconfirm the fact that HMPV infections detected by this combination approach represented genuine infections, we coated HMPV-infected LLC-MK2 cells onto slides for antibody detection using the immunofluorescence technique. All HMPV culture–positive patients who had serologic evidence of infection had a more than fourfold rise in antibody titers, and 15 patients seroconverted.

**Figure 4:**
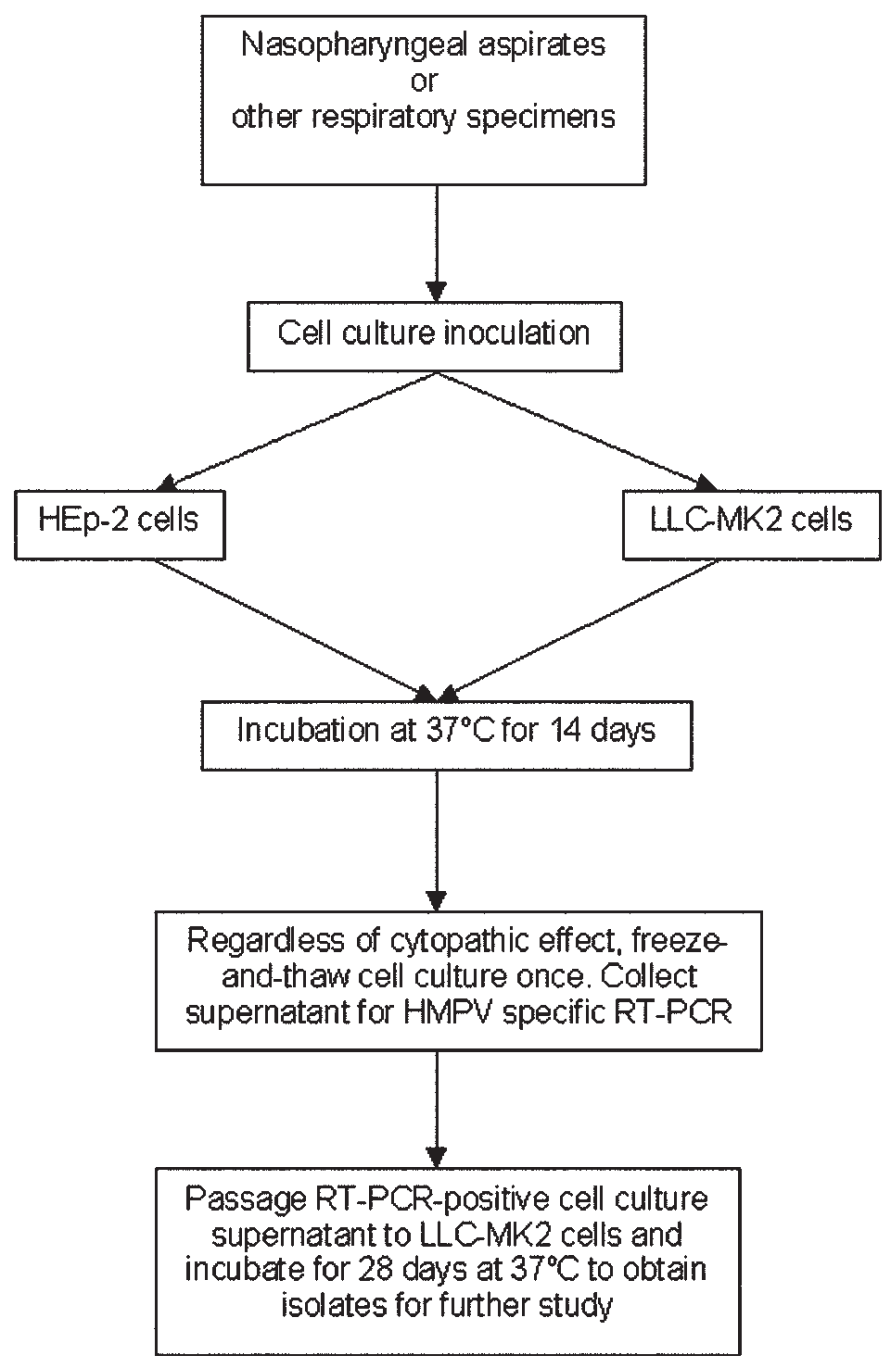
Combination approach of conventional virus isolation and molecular techniques to detect human metapneumovirus (HMPV) infection. Nasopharyngeal aspirates were examined in this study. This approach can be applied to other respiratory specimens. Prolonged incubation of rhesus monkey kidney **(**LLC-MK2) cells to 28 days for culture of original specimens may improve sensitivity of detection. Detection based on cytopathic effect is not sensitive for first-round culture from original specimens. All cell cultures should be examined by HMPV–specific reverse transcription-polymerase chain reaction. RT-PCR, reverse transcription-polymerase chain reaction.

Overall, our results indicated that the combination approach of using conventional virus isolation and molecular detection could be successfully applied to the isolation of HMPV (Figure 4). With this approach, we found that among the 48 study participants, 6 (12.5%) had both HMPV and coronavirus isolated from NPAs, 19 (39.6%) had HMPV, and 5 (10.4%) had coronavirus. Eighteen (37.5%) had no virus isolated from the cell lines that we used.

## Discussion

On the basis of a combination of conventional virus isolation system and molecular techniques, we found that 52.1% (25/48) of patients with SARS admitted to our hospital had HMPV infection. Isolation of HMPV is known to be difficult, which is why the virus could not be detected until recently. The first report on HMPV by van den Hoogen et al. (*1*) showed that the virus produced syncytia formation in tertiary monkey kidney cells, followed by rapid internal disruption of the cells and subsequent detachment from cell monolayer. The virus replicated poorly in Vero cells and human lung adenocarcinoma (A-549) cells and could not be propagated in MDCK cells or chicken embryo fibroblasts (*1*). In the study from Boivin et al. (*7*), multiple cell lines including LLC-MK2, HEp-2, MDCK, human foreskin fibroblast, Vero, Mink lung, A-549, human rhabdomyosarcoma (RD), transformed human kidney (293), and human colon adenocarcinoma (HT-29), were used for isolation of HMPV. The results showed that HMPV only grew on LLC-MK2 cells with cytopathic effect of round and refringent cells but without syncytia formation in most cases, an observation in agreement with our results. In that study, HEp-2 cell monolayers did not show cytopathic effect. Since the HEp-2 cells were not tested for HMPV RNA, we do not know whether our findings on HEp-2 cells were also observed by Boivin et al. In another study reported by Peret et al. (*6*), LLC-MK2, MDCK, and NCI-H292 cells were used; those researchers found that only LLC-MK2 cells produced cytopathic effect of focal rounding and without syncytia formation, which is also similar to our observation. The major difference in our approach for HMPV isolation compared to previous studies is the use of RT-PCR to enhance the detection of HMPV isolated from cell cultures. With this approach, we found that HEp-2 cells, a widely available and commonly used cell line, support the growth of HMPV. When RT-PCR was used to follow up all cell cultures, the sensitivity of HEp-2 cells was higher than LLC-MK2 cells, the cell line most commonly used in previous studies for HMPV. However, even using our approach, LLC-MK2 cells cannot be discarded, as in 12% of cases HMPV was only isolated from LLC-MK2 cells. In contrast, in the presence of HEp-2 cells, MDCK cells gave little additional value, as both specimens positive by MDCK cells had the viruses isolated from HEp-2 cells. In addition, our initial incubation of 14 days might not be optimal for isolating HMPV because Boivin et al. reported that the cytopathic effect took a mean incubation time of 17.3 days to develop (*7*). By prolonging the initial incubation of LLC-MK2 cells to 21 or 28 days, more HMPV infections might have been detected from our “negative” group.

Because all our study samples were collected from patients related to the outbreak of SARS that occurred in our hospital, one cannot simply infer that this in vitro growth property can be applied to all HMPV strains in general. Nevertheless, our approach of including HEp-2 cells, a widely available cell line, to search for HMPV, in particular for those cases related to SARS, needs to be considered. In our study, six patients were co-infected with HMPV and coronavirus. Although the number was limited, our findings suggest that HMPV and coronavirus have different in vitro tropisms, and the isolation of one virus does not affect the recovery of the other from different cell lines.

Overall, we confirmed that 25 (52.1%) of 48 patients admitted to our hospital with SARS had HMPV infections, with 6 also co-infected with coronavirus. However, the data on such high prevalence of HMPV should be interpreted cautiously. Our study population was based on persons and their family members who had been exposed in the index ward in our hospital. Thus, a co-circulation of two pathogens within our study group was possible. While the clinical presentations of all our study participants fulfilled the World Health Organization definition for a probable case of SARS (*13*), one should not infer, at this stage, that the prevalence of HMPV is similarly high in SARS outbreaks occurring elsewhere. On the other hand, the possibility of an important role of HMPV in the current worldwide outbreak of SARS should not be neglected. HMPV has also been detected in five of six SARS patients living in Canada (*15*). In that study series, coronavirus was also detected in five of six patients and four patients were co-infected with HMPV and coronavirus. A few recent studies implicate a strong association of a novel coronavirus with the worldwide outbreak of SARS (*16*–*18*). While both HMPV and coronavirus infections may result in severe respiratory tract diseases, their transmission efficiency may not be the same. This urgent question must be answered because the answer affects the priority for immediate development of control strategies.

During this outbreak of SARS, we have applied this combination approach of conventional virus isolation and molecular detection to establish the viral infection status of other patients hospitalized for SARS. We are in the process of analyzing a larger cohort to elucidate their clinical conditions, treatment responses, and epidemiologic links with respect to the infection status for both HMPV and coronavirus. Similar work in other parts of the world is needed.
